# PWC-ICA: A Method for Stationary Ordered Blind Source Separation with Application to EEG

**DOI:** 10.1155/2016/9754813

**Published:** 2016-06-02

**Authors:** Kenneth Ball, Nima Bigdely-Shamlo, Tim Mullen, Kay Robbins

**Affiliations:** ^1^Department of Computer Science, University of Texas at San Antonio, San Antonio, TX 78249, USA; ^2^Human Research and Engineering Directorate, U.S. Army Research Lab, Translational Neuroscience Branch, Aberdeen, MD 21001, USA; ^3^Qusp Labs, 6020 Cornerstone Court West, Suite 220, San Diego, CA 92121, USA

## Abstract

Independent component analysis (ICA) is a class of algorithms widely applied to separate sources in EEG data. Most ICA approaches use optimization criteria derived from temporal statistical independence and are invariant with respect to the actual ordering of individual observations. We propose a method of mapping real signals into a complex vector space that takes into account the temporal order of signals and enforces certain mixing stationarity constraints. The resulting procedure, which we call* Pairwise Complex Independent Component Analysis* (PWC-ICA), performs the ICA in a complex setting and then reinterprets the results in the original observation space. We examine the performance of our candidate approach relative to several existing ICA algorithms for the blind source separation (BSS) problem on both real and simulated EEG data. On simulated data, PWC-ICA is often capable of achieving a better solution to the BSS problem than AMICA, Extended Infomax, or FastICA. On real data, the dipole interpretations of the BSS solutions discovered by PWC-ICA are physically plausible, are competitive with existing ICA approaches, and may represent sources undiscovered by other ICA methods. In conjunction with this paper, the authors have released a MATLAB toolbox that performs PWC-ICA on real, vector-valued signals.

## 1. Introduction

Blind source separation (BSS), the process of discovering a set of unknown source signals from a given set of mixed signals, has broad relevance in the physical sciences. Independent component analysis (ICA) is a widely used approach to the BSS problem that seeks maximally statistically independent sources. Existing ICA algorithms can be broadly divided into two categories based on a definition of statistical independence and the corresponding optimization problem [1]. ICA by maximization of entropy is notably embodied by the Infomax [2], Extended Infomax [3], and Pearson [4, 5] ICA algorithms. Alternately, fixed-point algorithms such as FastICA [6] seek to maximize non-Gaussianity. Hyvärinen et al. [1] point out that these two perspectives are closely related, as the negentropy measure of non-Gaussianity used in FastICA and comparable algorithms has an information-theoretic interpretation in mutual information reduction that is fundamentally related to entropy maximization.

Standard applications of ICA to spatiotemporal signals such as EEG (electroencephalogram) treat each time point independently and do not use order information to separate sources. These traditional ICA models look for uncorrelated, statistically independent sources. While these ICA analyses have been highly successful in many applications, the fundamental assumptions of statistical independence do not necessarily fit with the view of the brain as a highly connected network of coupled oscillators. Motivated by work in dynamical systems using delay coordinates to reconstruct dynamics [7, 8], we explored methods to incorporate delay coordinates in ICA transformations.

We observe that given a discrete set of sequentially ordered vector observations, we can approximate the instantaneous rate of change by the time-scaled vector difference of consecutive pairs of observations. Furthermore, this rate of change closely corresponds to the sequential structure of the observed signals. Our approach is to map sequential pairs of observations (or, equivalently, their interpretation as a pair of approximate position and instantaneous velocity vectors) to a complex vector space, perform complex ICA, and map the results back to the original observation space. We demonstrate that a complex vector space is an attractive setting for ICA because it reduces the degrees of freedom of the problem relative to the sequential pair or tangent space interpretations in a way that preserves constraints on the demixing solution imposed by the assumption of stationarity in the underlying mixing problem. We refer to the resulting class of algorithms as* Pairwise Complex ICA* (PWC-ICA), reflecting the underlying mapping of sequential pairs of vector observations to complex space.

A central observation of the ICA algorithm evaluation reported by Delorme et al. [9] is that an ICA algorithm's ability to reduce component mutual information varies linearly with the fraction of components that fit single dipole sources. We make use of code and data made available by these authors to compare the performance of PWC-ICA in the EEG BSS paradigm of electric dipole sources. Because our approach seeks an ICA solution to the BSS problem in a complex setting, we do not expect and indeed do not find a comparable relationship between mutual information reduction and rates of effective dipole fitting.

The remainder of the paper is organized as follows.  Section 2 provides background, and Section 3 describes the PWC-ICA method.  Section 4 presents results of applying PWC-ICA to signals generated through various autoregressive models, with and without forward head modeling.  Section 5 evaluates the method on real EEG data, and Section 6 offers concluding remarks. Appendices are included to explicitly describe the models used to generate simulations in Section 4.

We demonstrate that by transferring the mutual information reduction (alternately maximization of non-Gaussianity) objective to the complex vector space we enable PWC-ICA to discover physiologically plausible sources of meaningful EEG activity that remain undiscovered by other ICA algorithms. We verify this by analysis of fitted dipoles on the real-world EEG data provided by Delorme et al. [9]. We also use the Source Information Flow Toolbox (SIFT) [10, 11] to generate simulated EEG data and analyze the discovered components in terms of correlation and distance to simulated sources.

## 2. Background

### 2.1. Independent Component Analysis and Applications

This section briefly describes the ICA model for blind source separation (BSS). Consider a sequentially ordered set of *n*-dimensional vector observations **x**(*t*) ⊂ *ℝ* ^*n*^. The ICA model assumes that *m* ≤ *n* statistically independent sources generate the observations. Each of the *m* sources at the *t*th observation corresponds to an element of the vector **s**(*t*) ∈ *ℝ* ^*m*^. Assume that the sources undergo linear instantaneous mixing by a linear transformation *ℝ* ^*m*^ → *ℝ* ^*n*^ specified by a full row rank matrix **A** ∈ *ℝ* ^*n*×*m*^ plus additive noise denoted by **ϵ**. The *t*th observation then satisfies **x**(*t*) = **A** **s**(*t*) + **ϵ**(*t*). For notational convenience, we specify successive operations by incrementing *t* by 1. In reality, the experiment has a sampling interval Δ*t* corresponding to the actual elapsed time between observations at *t* and *t* + 1. In other words, we reparameterize the signals by *t* ↦ *t*/Δ*t*, so that the new sampling interval is Δ*t* = 1.

ICA seeks an approximate demixing matrix **W** ∈ *ℝ* ^*m*×*n*^ that maximizes the statistical independence (via a variety of related optimization objectives) of the discovered sources. A projection formed by a subset of *m* rows of **W** corresponds to the Moore-Penrose pseudoinverse of **A** (or just the regular inverse when *m* = *n*), so that **W** **x**(*t*) = **W** **A** **s**(*t*) ≈ **s**(*t*) up to permutation. In the ideal case, *m* of the columns of **W** ^−1^ are *n*-dimensional vectors that we may interpret as the* independent components* (ICs). (See Hyvärinen et al. [1] for a comprehensive tutorial.)

The independent components discovered by ICA correspond to meaningful behavior in many applications, especially in the analysis of EEG data. Physiological and experimental evidence indicates that dipolar distributions of electric charge in the brain generate sources that may be recovered as ICA components. Dipole fitting algorithms [12] map the scalp field distributions associated with such components to spatial positions and moment orientations in the brain. ICA algorithms are also widely used to isolate artifacts within EEG data such as eye blinks, eye saccades, muscle activity, and other intermittent voluntary and involuntary biological processes that generate measurable electrical signal at the scalp [13–15].

### 2.2. Related Work

As stated briefly in the Introduction, one of the motivations for developing the PWC-ICA method is the observation that solutions to the optimization problem for many ICA algorithms are invariant with respect to ordering of the data. Given that the typical domain of the BSS problem consists of sequentially ordered observations, we believe that optimization objectives that take into account this ordering may result in useful solutions to BSS problems involving time series. However, this is not an original observation; we briefly review alternate solutions to the BSS problem that take into account the ordering of vector-valued signals.

#### 2.2.1. Second-Order Blind Identification

Second-order blind identification (SOBI) is an algorithmic approach to the BSS problem that jointly diagonalizes covariance matrices of the transformed data across a set of user-defined time intervals [16]. Thus, SOBI incorporates the ordering of data by considering the covariance of data across a set of predetermined lags. The term* second order* in SOBI refers to the use of a second-order statistics (variance) as the optimization criteria of the algorithm, as opposed to fourth-order methods (kurtosis) such as FastICA [6] or mutual information reduction methods such as Infomax [2]. The PWC-ICA approach proposed in this work is not second order from a statistics perspective but rather uses fourth-order statistics in a complex vector space.

#### 2.2.2. ICA of Autocorrelated Sources and Spectral ICA

Lee et al. [17] describe an ICA approach to functional magnetic resonance imaging (fMRI) data based on the spectral density of observations. The authors model sources in a BSS problem using autoregressive (AR) models and propose an ICA procedure that simultaneously seeks an optimal demixing matrix and parameters of the (stationary) AR process. They formulate an ICA procedure in the frequency domain and use linearity of the discrete Fourier transform to find the demixing matrix.

In addition to the work of Lee et al. [17], several other authors have proposed ICA paradigms that model sources using linear AR models and/or perform ICA in the frequency domain [18–21].

#### 2.2.3. Complex BSS via the Hilbert Transform

Hirayama et al. [22] recently proposed a method that simultaneously combines a BSS problem framed in complex space and connectivity clustering of EEG or magnetoencephalographic (MEG) signal data. The proposed method maps real-valued signal data to a complex vector space by taking the real part of the complex vectors to be the original signals and then adding the Hilbert transform of the signal vectors as the imaginary part of the complex vectors to generate the analytic signal. The authors then solve a BSS problem in the complex space based on a problem-specific optimization objective that includes a notion of clustering components corresponding to particular states.

The Hilbert transform is linear and hence commutes with linear mixing/demixing transformations when applied componentwise to a vector series. Thus, Hirayama et al. [22] are able to find an optimal real-valued demixing matrix **W**, along with parameters specific to the connectivity model, while incorporating the complex analytic signal in their optimization objective. In contrast, our approach first specifies an alternative method of mapping real signals to a complex space, motivated by the instantaneous rate of change of signals. Next, we find a complex demixing matrix (representing a change of basis in the complex signal space, i.e., a solution to the chosen complex ICA optimization paradigm), and finally we interpret this demixing operation as a real linear transformation mapping multidimensional signals to sources.

While a description and comparative implementation of the full approach of Hirayama et al. [22] is beyond the scope of the present paper, the use of a Hilbert transform to derive complex analytic signals for subsequent analysis is of interest. Specifically, the complex analytic signal may be derived via a Hilbert transform and then fed through the subsequent steps of the PWC-ICA workflow. We include an example of this approach (labeled “Hilbert complex FastICA”) for illustrative purposes in our results. Our performance comparisons indicate that the complex signals derived in PWC-ICA seem to encode interesting dynamics of the observed system that may not be included in the complex analytic signals.

## 3. PWC-ICA for Order-Based Source Separation

Motivated by the observation that ordering and timescale are intrinsic properties that are likely to be useful in source analysis of vector-valued time series, we introduce the PWC-ICA method for blind source separation. Let **x**(*t*) be an *n*-dimensional vector-valued time series that can be expressed as a linear mixture of independent sources, **s**(*t*):

(1) $$\begin{array}{c} x\left(\;t\;\right)=As\left(\;t\;\right). \end{array}$$

If the mixing matrix **A** is independent of time, then the rate of change of **x**(*t*) with respect to time satisfies

(2) vtx˙t=ddtxt=ddtAst=Addtst=As˙t,

provided that the signals and sources are differentiable. In the language of differential geometry, we can think of (**x**(*t*), **v**(*t*)) as the tangent bundle for **x**(*t*). The* pushforward* of the mapping of **A** to this tangent bundle is simply

(3) $$\begin{array}{c} \left[\begin{array}{c} x\left(t\right)\\ v\left(t\right) \end{array}\right]=\left[\begin{array}{cc} A & 0\\ 0 & A \end{array}\right]\left[\begin{array}{c} s\left(t\right)\\ \dot{s}\left(t\right) \end{array}\right]. \end{array}$$

We seek solutions (demixing matrices) of the form

(4) $$\begin{array}{c} {\left[\;\begin{array}{cc} A & 0\\ 0 & A \end{array}\;\right]}^{-1}=\left[\begin{array}{cc} A^{-1} & 0\\ 0 & A^{-1} \end{array}\right]=\left[\begin{array}{cc} W & 0\\ 0 & W \end{array}\right], \end{array}$$

where **W** ^−1^ = **A**.

The formulation assumes smoothness of the signals and imposes stationarity on the mixing matrix. For this reason, we refer to this formulation as* stationary source separation*.

It is not immediately clear how to solve the stationary mixing problem directly in *ℝ* ^2*n*^ with the proposed constraints (i.e., finding **W** that simultaneously minimizes mutual information among components in both the base and tangent space). The approach taken in this work is to map the dynamic signals in *ℝ* ^2*n*^ to the complex space *ℂ* ^*n*^ by associating the real vector (**x**(*t*), **v**(*t*)) with complex vector, **x**(*t*) + *i* **v**(*t*). A complex linear transformation, Φ, on *ℂ* ^*n*^ may be represented (the representation is not unique but induced by the canonical complex linear structure $J=\left[\begin{array}{cc} 0 & -I\\ I & 0 \end{array}\right]$ on *ℝ* ^2*n*^≅*ℝ* ^*n*^ × *ℝ* ^*n*^; linear transformations on *ℝ* ^2*n*^ that commute with **J** are also complex linear transformations on the associated complex vector space *ℂ* ^*n*^) in *ℝ* ^2*n*^ as

(5) $$\begin{array}{c} \left[\begin{array}{cc} R\left(\Phi \right) & -I\left(\Phi \right)\\ I\left(\Phi \right) & R\left(\Phi \right) \end{array}\right]. \end{array}$$

Here *ℜ*(Φ) and *ℑ*(Φ) are the real and imaginary parts of Φ. Notice that (5) has equal diagonal blocks. Thus, in order to find ICA demixing solutions that match (4), we need to minimize the contribution of the off-diagonal term *ℑ*(Φ) to the demixing solution.

The optimization objectives of ICA methods are generally invariant with respect to permutations and nonzero scale transformations of the resultant independent components. Complex ICA algorithms with optimization objectives that depend on the moduli of observations (including the version of complex FastICA we choose to use in this paper) have an additional invariance in the optimization objective with regard to phase shifts of the complex independent components. This phase shift invariance provides a mechanism for minimizing the contribution of the imaginary (off-diagonal) terms in the complex demixing solution.

Let *ϕ*_*j*_ refer to a phase shift in the *j*th independent component. Due to the phase invariance of the complex independent components, Φ exhibits nonuniqueness up to multiplication by the diagonal matrix with values *e* ^*iϕ*_*j*_^ for each of the *j*th diagonal entries, prompting us to make the identification:

(6) $$\begin{array}{c} \Phi ≅diag\left[\;e^{i\phi_{1}},e^{i\phi_{2}},\ldots ,e^{i\phi_{n}}\;\right]\cdot \Phi , \end{array}$$

for an arbitrary set of real phase shifts {*ϕ*_1_, *ϕ*_2_,…, *ϕ*_*n*_}. This identification can be expressed blockwise in the *ℝ* ^2*n*^ setting as

(7) diageiϕ1,eiϕ2,…,eiϕn·Φ⟼C−SSCRΦ−IΦIΦRΦ=CRΦ−SIΦ−SRΦ−CIΦSRΦ+CIΦCRΦ−SIΦ,

where *C* and *S* are *n* × *n* diagonal matrices with *j*th diagonal entries cos⁡(*ϕ*_*j*_) and sin⁡(*ϕ*_*j*_), respectively. We select the PWC-ICA demixing matrix **W** on the original space to be the diagonal block term of the demixing matrix in the complex space: **W** = *Cℜ*(Φ) − *Sℑ*(Φ); and we seek a set of phase shift values {*ϕ*_1_, *ϕ*_2_,…, *ϕ*_*n*_} that maximizes the contribution of the diagonal term, **W**, while minimizing the off-diagonal term ±(*Sℜ*(Φ) + *Cℑ*(Φ)).

We accomplish this by formulating an optimization objective of maximizing the source component variances generated by **W**. Let **s**(*t*) = **W** **x**(*t*) denote the (real) vector of approximated sources in terms of the signal vector **x** at time *t*. Then the variances of the (zero-mean) components **s** over the time course of the analyzed signal are given by the* diagonal* values of the matrix:

(8) 1NWxxTWT=1NCRΦx−SIΦx·xTRΦTC−xTIΦTS.

Recall that *S* and *C* are defined to be diagonal matrices and are invariant under the transpose operation and *N* is the number of samples/observations in the multidimensional signal **x**. Differentiation with respect to each phase shift yields critical points of each diagonal value at

(9) $$\begin{array}{c} \phi_{j,\text{critical}}=\frac{1}{2}\arctan\left(\;\frac{2{\left(R\left(\Phi \right)x\right)}_{j}{\left({\left(I\left(\Phi \right)x\right)}_{j}\right)}^{T}}{{\left(I\left(\Phi \right)x\right)}_{j}{\left({\left(I\left(\Phi \right)x\right)}_{j}\right)}^{T}-{\left(R\left(\Phi \right)x\right)}_{j}{\left({\left(R\left(\Phi \right)x\right)}_{j}\right)}^{T}}\;\right)+\frac{k\pi }{2}, \end{array}$$

for integer values of *k*. Subscripts on matrices in the above equation indicate the row of the matrix. Manipulation by trigonometric identities reveals that the variance is a positive constant added to a sinusoidally oscillating function of 2*ϕ*_*j*_. Therefore, comparing variances at the critical phase values for *k* = 0 and *k* = 1 yields the phase shift associated with a local maximum in variance. The critical phase shift solutions are *π* periodic, yielding 2 maximizing values within an interval [0,2*π*). Both solutions yield equivalent answers in the context of the ICA problem, as they correspond to multiplying the discovered basis vector associated with the *n*th ICA component by ±1.

With the set of *n* phase shifts maximizing variance of the PWC-ICA components, we define the real demixing matrix to be

(10) $$\begin{array}{c} W={\left.\;C\;\right|}_{{\left\{\phi_{j,\text{critical}}\right\}}_{j=1}^{n}}R\left(\;\Phi \;\right)-{\left.\;S\;\right|}_{{\left\{\phi_{j,\text{critical}}\right\}}_{j=1}^{n}}I\left(\;\Phi \;\right). \end{array}$$

Interestingly, phase shifts that maximize the variance of variables **W** **x** also* minimize* the variance of the variables that are generated by the imaginary part of diag[*e* ^*iϕ*_*j*,critical_^] · Φ, which can be easily verified by repeating the analysis above for the imaginary part. In our *ℝ* ^2*n*^ interpretation of the transformed pairwise space, this is equivalent to minimizing the contribution of cross-terms from the tangent to base space, and vice versa. The difference between these two variances may serve as a heuristic for evaluating the quality of fit of components specified by the real demixing matrix.

Rodriguez et al. [23] suggest a similar resolution to the problem of phase shift invariance in dealing with complex-valued ICA on fMRI data: maximizing the variance of the real parts of discovered complex components. Their approach involves taking the first principal component from each complex independent component treated as a vector in *ℝ* ^2^. Our analytic solution to finding phase shifts does not rely on PCA.

**Figure 1:**
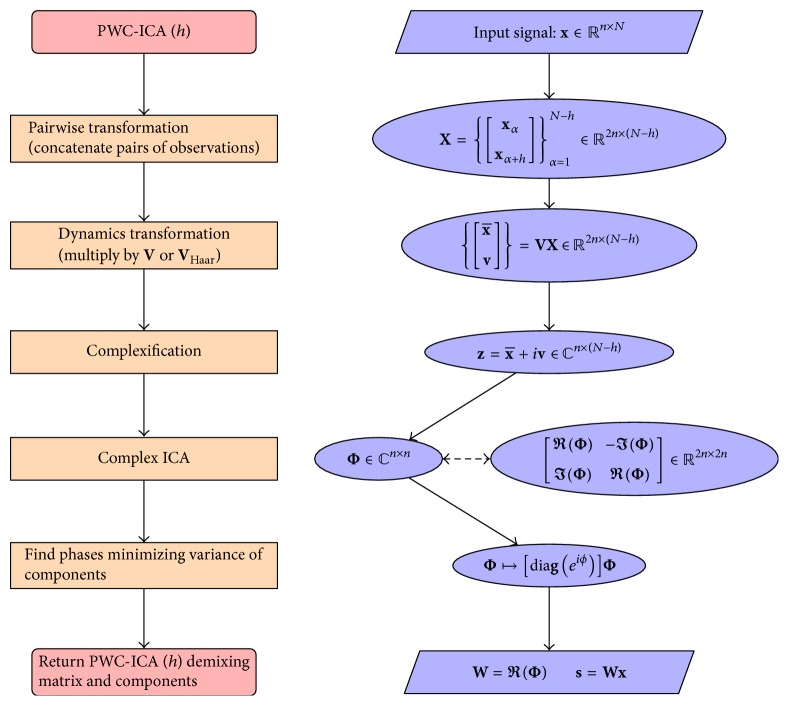
Flow chart summarizing the PWC-ICA algorithm. The left column depicts the conceptual progression of the algorithm proceeding from top to bottom, whereas the right column summarizes the central mathematical operation at each step. Note that the two side-by-side bubbles connected by a dashed line at the complex ICA step illustrate the parallel interpretation between the dynamic state of the signal and the complexification of the phase space. The operators **V** and **V**_Haar_ indicate the linear transformation from the pairwise space to the dynamic phase space. Arrays $\overset{-}{x}$ and **v** are the “base” and “velocity” components of the pairwise vectors in **X** transformed to the dynamical phase space. The array **z** represents the complexification of the phase space vectors, Φ is the complex ICA demixing matrix, and **W** is the PWC-ICA (*h*) demixing solution.

To summarize, our general strategy is to map the source separation problem from *ℝ* ^*n*^ to the tangent bundle in *ℝ* ^2*n*^, a process that implicitly imposes additional smoothness requirements on the sources and time-invariance constraints on the mixing process. We then further transform the problem in *ℝ* ^2*n*^ to *ℂ* ^*n*^ and solve an equivalent complex source separation problem. Finally we use the phase shift invariance of the complex solution to choose a phase that produces an optimal match to a demixing solution of the form given by (4). We call this approach* Pairwise Complex ICA* (PWC-ICA). We summarize the PWC-ICA algorithm in the flow chart in Figure 1. The next subsection provides more details about different approaches to approximating the tangent bundle. The following subsection discusses the complex ICA methods used for the implementation.

### 3.1. Mapping Real Signals in Complex Space

In this section, we discuss the mapping of real-valued signals first into a dynamic phase space and then into a complex vector space. Suppose a set of discrete signal observations consists of *N* vectors **x**(*t*) acquired at a constant sampling rate. We can form a new* pairwise* set of *N* − 1 vectors by identifying **x**(*t*) with the 2*n*-dimensional vector (**x**(*t*), **x**(*t* + 1)), analogous to the idea of a delay embedding in dynamical systems. From these pairs of signal observations, we define a linear transformation *V* : *ℝ* ^2*n*^ → *ℝ* ^2*n*^ that incorporates approximate rate of change information into one-half of the 2*n* components in the transformed vectors. Let *V* be the transformation:

(11) Vxt,xt+1Vxtxt+1=I2I2−IΔtIΔtxtxt+1=xt+xt+12xt+1−xtΔt≈xt+12vt+12.

The first *n* components of the transformed pairwise vectors approximate the signal at times *t* + 1/2, and the second *n* components approximate the rates of change at times *t* + 1/2. The notation implies a time step determined by the sampling frequency.

The above transformation associated with the matrix **V** is similar to that used in the Haar wavelet, except in this instance we include a time scale factor and do not downsample the signal [24]. In fact, if we were to disregard the physical interpretation of the difference vector as an estimation of the instantaneous rate of change, we could use the linear transformation associated with

(12) $$\begin{array}{c} V_{\text{Harr}}=\frac{1}{\sqrt{2}}\left[\begin{array}{cc} I & I\\ -I & I \end{array}\right], \end{array}$$

the 2*n* × 2*n* Haar matrix associated with the Haar wavelet. In this case, the summed and differenced vectors correspond to the approximation and details of the wavelet transformation, respectively. The transformations **V** and **V**_Harr_ differ only in the scaling of their respective components. The **V** transformation incorporates time scale information and represents physical position and velocity. On the other hand, **V**_Harr_ is an orthogonal transformation. The variance of randomly ordered Gaussian distributions is invariant with respect to transformations by **V**_Haar_, implying that the noise in the mixed **v** (induced by **ϵ** in the original space) should have the same distribution as **ϵ** for **V**_Haar_.

We have described two closely related approaches to transforming a pairwise embedding of a sampled signal to a series of samples in a dynamic phase space. Both of these transformations implicitly incorporate time scale information because successive time points are spaced at the sampling interval. We can change the time scale by using mappings of the form (**x**(*t*), **x**(*t* + *h*)) for integer values *h*. The transformation effectively estimates the signal and its average rate of change on the time scale *h*. In general, we refer to the transformation **V** lagged by integer value *h* as **V**_*h*_ and the corresponding algorithm as PWC-ICA (*h*).

A multiscale version can also be implemented using the matrix $V_{\text{Harr}}=\left(1/\sqrt{2}\right)\left[\begin{array}{cc} I & I\\ -I & I \end{array}\right]$ as a linear operator on the concatenated 2*n*-dimensional vectors of time-lagged pairs of observations. However, in this case we can no longer interpret the overall transformation as convolution by a Haar wavelet. Instead, the sums and differences are the result of convolution of *h*-sized windows of the components of signal data vectors with the wavelets $\left[\begin{array}{ccccc} 1 & 0 & \cdots & 0 & 1 \end{array}\right]$ and $\left[\begin{array}{ccccc} -1 & 0 & \cdots & 0 & 1 \end{array}\right]$, respectively. For simplicity, we will continue to refer to the transformation on pairwise vectors as **V**_Haar_, while noting that the resulting overall transformations on signals are not truly the result of a Haar wavelet convolution process.

### 3.2. Complex ICA

We tested several existing complex ICA algorithms, including the method of Entropy Bound Minimization (EBM) [26] and robust ICA [27], before selecting an extension of FastICA to the complex case originally proposed by Bingham and Hyvärinen [28]. For the present analysis, we use the MATLAB function “FicaCPLX,” described by Koldovský and Tichavský [29], itself an extension of the complex FastICA algorithm. Alternate approaches may result in different PWC-ICA features; for instance, EBM is capable of finding both circular and noncircular complex sources, as well as sub- and super-Gaussian sources in the complex setting. On the other hand, the FastICA algorithm implemented by Koldovský and Tichavský [29] was stable and fast, performing well on EEG data in our target domain.

The ICA model implemented by the FastICA extension to complex vector spaces assumes that the underlying sources are complex random variables with zero-mean, unit variance, and uncorrelated real and imaginary parts of equal variance. Bingham and Hyvärinen [28] point out that this condition is expressible in terms of covariance and pseudo-covariance matrices: *E*[**s** **s** ^*H*^] = **I** and *E*[**s** **s** ^*T*^] = 0, where **s** is a matrix with column vectors **s**(*t*) ∈ *ℂ* ^*n*^ representing the complex sources at time *t*, the superscript *H* indicates the conjugate transpose, *E*[·] is the expectation operator, and 0 refers to the zero-valued matrix. Consequently, a necessary preprocessing step for complex FastICA is signal whitening so that *E*[**z** **z** ^*H*^] = **I**. We therefore perform an intermediate whitening transformation on **z** using a complex whitening matrix **S**_*ℂ*_ of the form

(13) $$\begin{array}{c} S_{C}={\left(\;\sqrt{cov\left(z^{*}\right)}\;\right)}^{-1}. \end{array}$$

Here the square root symbol denotes the matrix square root, and cov(·) represents the covariance matrix of the vector-valued signals. This formula is a complex version of the whitening approach used in various ICA implementations, including those found in EEGLAB [30]. We subsequently derive a complex-valued linear transformation Φ that decomposes the whitened signals (**S**_*ℂ*_ **z**) into mutually independent complex sources according to the FastICA objective. To simplify the notation, we assume the complex ICA demixing matrix acting on complex signals incorporates the whitening transformation and express Φ**S**_*ℂ*_ simply by Φ, according to context.

## 4. Experiments on Simulated Sources

In this section, we explore the performance of PWC-ICA on simulated data. To simulate EEG acquired from coupled networks of brain sources, we used coupled networks of autoregressive (AR) models generated using the Source Information Flow Toolbox (SIFT) [10, 31]. We ran comparative tests of PWC-ICA and existing ICA approaches on signal data generated by both random mixings and realistic forward models. In Section 4.1, we simulate sources using vector AR (VAR) models, first without coupling and then with static and dynamic coupling. We randomly mix sources into signals and observe how well the demixing matrix of each BSS algorithm matches the original mixing matrix. In Section 4.2, we generate source data via dynamically coupled AR sources and apply a realistic forward head model to mix the sources, resulting in a physical model of EEG scalp signals.

The explicit equations for all of the simulations presented in this section are included in Appendix A for reference. Further, the exact simulated data used for the present analyses (as well as the means to generate similar datasets) and scripts to perform the analyses are available as a MATLAB toolbox at https://github.com/VisLab/pwcica-toolbox.

### 4.1. Performance on Randomly Mixed Sources

To compare the performance of PWC-ICA algorithms with AMICA [32], Extended Infomax [2, 3], and FastICA [6], we analyzed sources based on vector AR models of both uncoupled and coupled harmonic oscillators, as well as simple statically coupled sources. In each experiment, we randomly generated a fixed number of well-conditioned mixing matrices, mixed the simulated sources, and recovered demixing matrices based on each BSS algorithm.

We evaluated the accuracy of each demixing matrix **W** at reconstructing sources mixed by **A** (up to scale and permutation) using the Amari index [33], a measure of how closely the product **P** = **A** **W** approximates a generalized permutation matrix, of which the identity matrix is a special case. In other words, the Amari index generalizes the notion of **W** being the matrix inverse of **A**, allowing for permutations and scale transformations of the column vectors of **W** ^−1^ when compared to the actual mixing matrix **A**. For an *n* × *n* matrix **P** = (*P*_*ij*_), the Amari index is defined as

(14) Amari index=12nn−1∑i∑jPijmaxk⁡Pik−1+12nn−1∑j∑iPijmaxk⁡Pkj−1.

An Amari index of zero for a nonsingular matrix **P** indicates that **P** is a permutation of a diagonal matrix, while an index of one indicates that the entries of matrix **P** are the same constant. Thus, a lower value of the Amari index indicates that **W** is better at isolating the individual sources.

The scale factor 1/(2*n*(*n* − 1)) assures that the Amari index has a range [0,1] regardless of the dimension *n*. This scale factor is not explicit in Amari et al. and other papers using the Amari index. However, in this case the scaling is appropriate because the experiments described below were generated using different values for the dimension.

We selected the Amari index for its easy and frequent use in ICA performance studies. However, Ilmonen et al. [34] report that there might be pitfalls in comparative studies using the Amari index due to dependency on model formulation. They suggest an alternate performance index for ICA. We also implemented this alternative index, evaluated the BSS solutions using the new index, and performed the same statistical analysis as reported in Table 1. We found no change in the patterns of statistical significance reported in Table 1 for this alternative performance index.

Since computation of the Amari index requires* a priori* knowledge of the mixing matrix, we performed three simulations experiments of varying complexities in order to compare the performance of PWC-ICA to other algorithms. All models were based on vector autoregressive (VAR) models provided in the Source Information Flow Toolbox (SIFT) MATLAB package [31].  Experiment 1 used ten uncoupled oscillators (the sources).  Experiment 2 consisted of a network of five general AR sources linked by constant coupling strengths.  Experiment 3 consisted of a network of ten oscillators whose coupling strengths varied in time. Appendix A provides full details and equations for all three models. In each experiment, we generated 100 epochs of 500 time points at a sampling rate of 200 Hz and included randomized damping coefficients as well as Gaussian noise in the generative model at a 1 : 1 ratio.

Table 1 summarizes the resulting mean Amari indices across 20 repetitions of each of three simulation experiments described in more detail in Appendix A. For each experiment, we report the average of the Amari index values for each experiment. The results varied little across repetitions of the experiments, with the maximum standard error of the mean (SEM) being less than 0.014 in all cases. Recall that PWC-ICA (*h*) refers to transformations of pairs of observations (separated by a time interval *h*Δ*t*). The PWC-ICA method subsequently maps these pairs to the real and imaginary parts of a complex signal vector. PWC-ICA (*h*) Haar refers to transformations that ignore the sampling interval in the formation of the vectors. Table 1 also includes the results from an alternative complex ICA representation based on the Hilbert transform combined with complex FastICA as described in Hirayama et al., as well as a randomly generated baseline. The baseline was computed by generating 50,000 random, nonsingular, demixing matrices for each of the 20 mixing matrices and computing the Amari index in each case. The rationale behind this baseline is that it is the expected value of the Amari index when the demixing matrix is chosen at random.

Bold values indicate methods that significantly outperformed AMICA, Extended Infomax, and FastICA for a particular model class. The *p* values were computed using a Welch's paired *t*-test. We observe significantly better performance (as measured by the Amari index) for time-invariant PWC-ICA approaches relative to AMICA, Extended Infomax, and FastICA for Experiments 1 and 3. This was also the case for the Hilbert transform method, which is also a time-invariant mapping. In Experiment 2, none of the tested PWC-ICA algorithms significantly outperformed all three AMICA, Extended Infomax, and FastICA algorithms. In both Experiments 1 and 3, the sources were generated by a simple harmonic oscillator model. The results indicate that the rate of change components of PWC-ICA may be better able to distinguish this time varying behavior.

In Experiment 1, PWC-ICA (2) Haar and PWC-ICA (4) Haar also outperformed the Hilbert transform approach (statistically significant according to a Welch's paired *t*-test with *p* values 1.05*E* − 12 and 1.34*E* − 8, resp.). Recall from Section 2.2.3 that the complex Hilbert method described here generated complex analytic signals via the Hilbert transform and then fed those complex signals through the remaining PWC-ICA workflow by training a complex FastICA model on those analytic signals and returning the real part of the complex demixing matrix (with phase transforms that maximized component variance).

The preceding analyses examined the behavior of PWC-ICA approaches relative to AMICA, FastICA, and Extended Infomax in simulations where the sources underwent random well-conditioned mixing. The results reveal distinctions among different approaches and suggest that incorporating dynamic information can be useful. In the next section, we examine the behavior of these algorithms when simulated data is mixed according a realistic head model typically found in the ICA analysis of EEG data.

### 4.2. Forward-Projected Sources Using a Realistic Head Model

**Figure 2:**
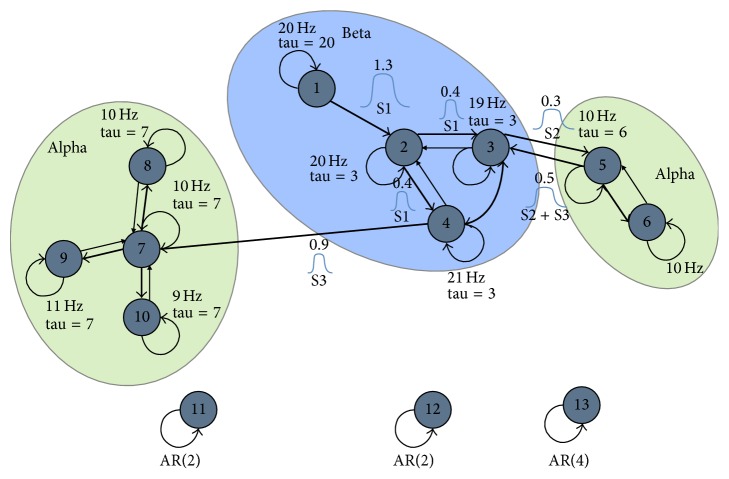
A schematic of a hypothetical seizure model using a vector autoregressive (VAR) model incorporating 13 dynamically coupled sources. Reproduced with permission from Mullen [25].

In this section, we evaluate simulations based on more realistic head modeling using a simulation reported by Mullen [25] and included with SIFT. We selected this model, graphically depicted in Figure 2, because it provides a generative model for dynamic coupling that can be associated with a physical forward head model.

The model, which is described in more detail in Appendix B, consists of 13 coupled damped oscillator sources described by time varying vector autoregressive models (VAR) of order 6. Model sources 1–10 are damped harmonic oscillators clustered into three dynamically coupled groups, while sources 11–13 are uncoupled throughout the experiment. The simulation proceeds in three stages (S1, S2, and S3), with the activity initially localized in the beta cluster and proceeding to each of the alpha clusters successively. Inter- and intracluster coupling is modeled using dynamic, cross-component AR coefficients that vary according to generalized Gaussian waveforms of the labeled amplitude centered at each of the stages.

To generate 64-channel EEG data, we used SIFT routines to model the sources as dipoles randomly selected from an atlas of physically plausible locations on the cortical surface. Source activations were projected through a three-layer Boundary Element Method (BEM) forward head model derived from the MNI “colin27” brain [35], and sensor noise was introduced at a signal-to-noise ratio of 1 : 1. We applied the various BSS algorithms to the resulting 64-channel scalp headset data and recovered 64 components. Ideally, a solution to the BSS problem should recover the individual sources as the activations of individual components. With* a priori* knowledge of the sources, we can measure how well at least one component in each algorithm recovers each source by finding the best correlations between the component and source time courses.

**Figure 3:**
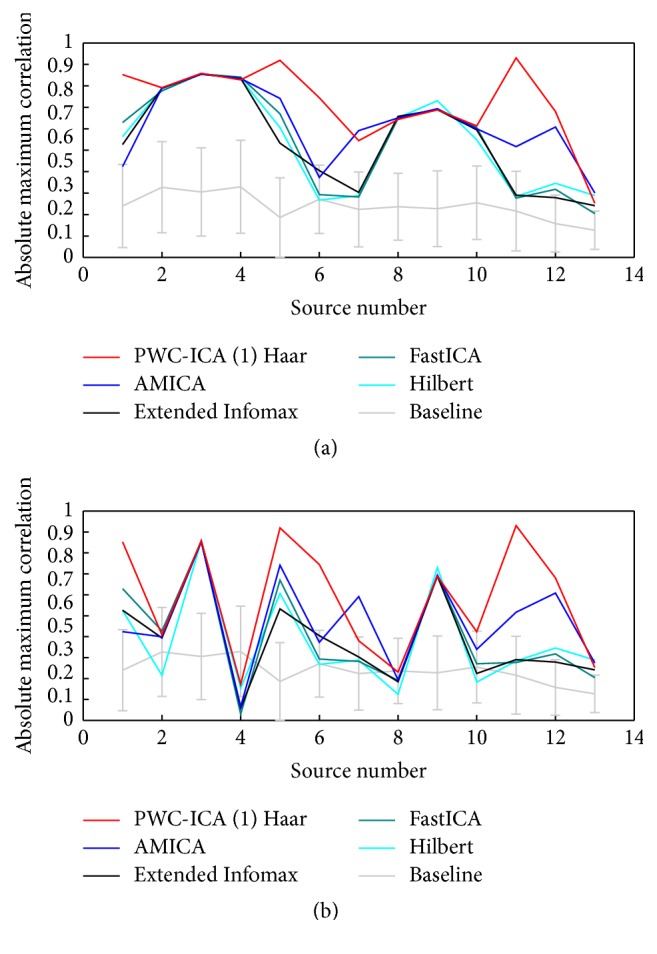
Plot of the best absolute correlation value between some component and each source for several BSS algorithms. (a) For each source, the plotted value is the highest absolute correlation attained between at least one component from the particular algorithm and that source. (b) The highest absolute correlation between any source and component is plotted and then removed from subsequent comparisons. The baseline measure in both plots is the average of the absolute correlations between all 64 generated signals and the source. The error bars are the standard deviations of those correlations.

Figure 3(a) shows a plot of the (absolute value) of the best correlations by source for several algorithms for generating 64 source components. For each of the 13 simulated sources, we tested the correlations between all 64 components and that source. We report the highest magnitude correlation for that source, irrespective of dipole location. Thus, the plot in Figure 3(a) shows how well at least one component of various methods recovered the time course of each source.

In the remaining simulation experiments, we report results for the PWC-ICA (1) Haar algorithm, which seems to perform better than other PWC-ICA approaches within the chosen simulations. We observe that PWC-ICA (1) Haar correlates with a given source at least as well as AMICA, Extended Infomax, and FastICA. In the case of the sources 1, 5, 6, and 11, PWC-ICA (1) Haar has better correlations than the alternative approaches. Because PWC-ICA seeks a transformation that minimizes mutual information (or maximizes non-Gaussianity) in a complex space that incorporates dynamics, it may be able to separate meaningful nonindependent sources, depending on the chosen time-offset used to map to the complex vector space.

Palmer and Makeig [36] demonstrate that basic ICA (in the case of Infomax) may be sufficient to separate independent subspaces without necessarily solving the subproblem of separating the less or nonindependent components within an independent subspace. In fact, the ISA approach proposed by Casey and Westner [37] may be more similar to the initial steps of PWC-ICA, in the sense that both approaches leverage a dynamic transform to augment the ICA approach in a higher dimensional space.

Because the simulation model described in Figure 2 arranges sources into dynamic clusters (which may be regarded as partially independent subspaces of the source space) and the resulting time courses of those coupled sources are somewhat correlated, we suspected that these algorithms were discovering single components that correlate well with multiple sources from the same cluster. We thus also examined an alternative* greedy* approach for determining the best correlation between components and a source. Instead of simply finding the most highly correlated component for each source, we find the absolute best correlation among all source/component pairs and remove that component and source from consideration before finding the next best source/component combination. We proceed in this manner until all 13 sources have been identified with a* unique* component.

Figure 3(b) displays the results of the greedy analysis. This greedy approach indicates that some discovered components correlate highly with multiple sources. We also note that the relatively good PWC-ICA performance on sources 1, 5, 6, and 11 is preserved under the greedy correlation analysis, further indicating that in this simulated experiment PWC-ICA (1) Haar does find components undiscovered by AMICA, Extended Infomax, and FastICA that correlate well with real source behavior.

We also performed a correlation analysis on simulated data for the three models of Section 4.1 and found the resulting correlations were consistent with the observed performance measured by Amari index. Our results are reported in Table 2.

### 4.3. Fitting Dipoles to Realistic Forward Model Components

The previous section compared the correlation between discovered components and actual sources using simulated sources with a realistic forward head model. This section examines the extent to which the dipoles computed from these components recover the original spatial locations of the sources. The 13 simulated sources are modeled as electric dipoles spatially distributed throughout a brain, and the observed signals are generated by mapping those sources via a linear transformation (forward model) to 64 electrodes spatially distributed on the scalp of the head model. If an algorithm truly separates these dipole sources, we can fit each component to recover the original dipole location and orientation as discussed in more detail in Section 5. With prior knowledge of the spatial distribution of the original sources, we can compare the actual source locations with the locations of the best-fit dipole for each component discovered by an ICA algorithm such as FastICA, Infomax ICA, or AMICA or by a PWC-ICA algorithm.

We use the dipole fitting routines found in the DIPFIT 2.3 plugin for EEGLAB [12, 30, 38] in the subsequent analysis. Specifically, we map each component to a best fitting dipole using a spherical four-shell head model with a total head radius of 85 mm.

**Figure 4:**
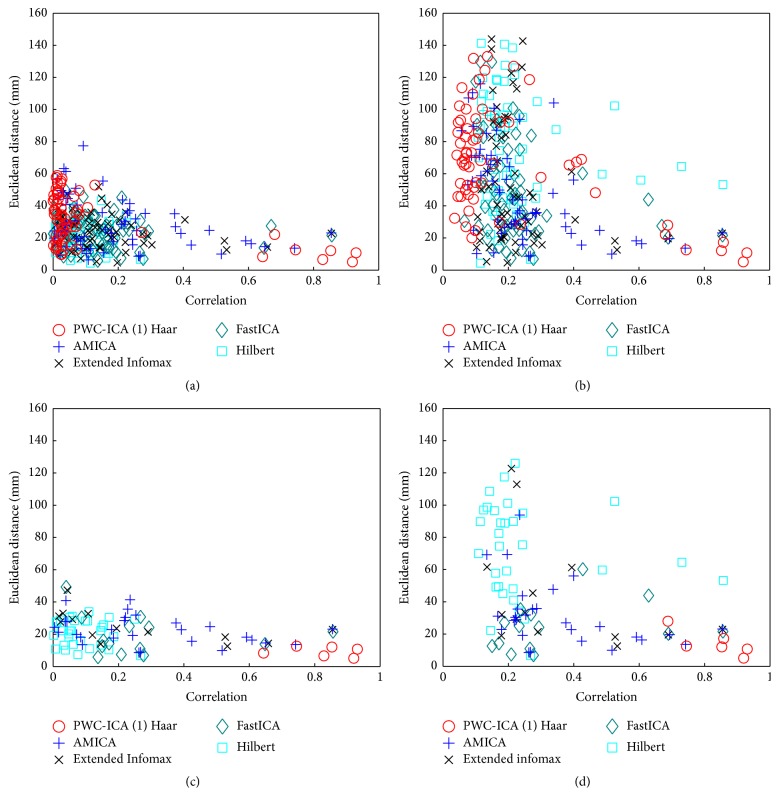
Plots of distance versus absolute time correlation between the dipoles corresponding to calculated components and the closest source. (a) Closest source to a component was determined by spatial distance between the associate dipoles. (b) Closest source to a component was determined by maximum absolute time correlation. Plots of distance versus absolute time correlation between physically plausible fitted dipoles with residual variance (RV) less than or equal to 15% and the closest actual source (c) by distance and (d) by absolute time correlation for different BSS algorithms.

In order to examine the extent to which the spatial locations of the fitted dipoles (especially those that correlated well with a source) matched the known locations of the 13 sources, we ran two analyses. First, we found the source closest in Euclidean distance to each component's fitted dipole and then plotted this distance against the absolute correlation between the corresponding time course of the component and the source. Second, we found the most highly correlated source for each component and compared the Euclidean distance between that component's fitted dipole and this source. Figures 4(a) and 4(b) show the results of both analyses for several methods. Figures 4(c) and 4(d) show the corresponding analysis, when only components that fit dipoles with residual variance less than 15% were considered.

Each BSS algorithm discovers 64 components, which approximate only 13 unique sources. Because the BSS problem is overfit when trained on the entire 64-dimensional dataset, the algorithms find many components that correspond to physically plausible dipoles but* do not* correlate well with the known sources as shown in Figures 4(a) and 4(b). By physically plausible we mean that the dipoles were spatially located in or around the head and the corresponding scalp maps fit to those dipoles with relatively low residual variances.

Figures 4(c) and 4(d) show a striking difference between PWC-ICA (1) Haar and the traditional AMICA, Extended Infomax, and FastICA algorithms. While the traditional algorithms produce many more “physically plausible” dipoles, many of these dipoles show little relationship to actual sources. On the other hand as shown by Figure 4(d), PWC-ICA (1) Haar only produces 6 plausible dipoles, but they are close both in time and space to the actual sources.

**Figure 5:**
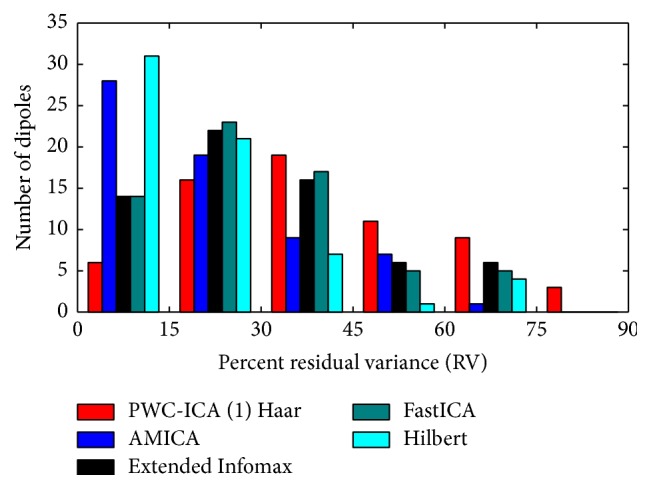
Histogram illustrating the distribution of residual variance (RV) percentages for different algorithms. Lower RV percentages indicate a better solution to the dipole fitting optimization objective that is derived from the physical location of electrodes on the scalp and the BSS solution's demixing matrix.

Figure 5 illustrates the distribution of residual variance of dipoles for the 64 components discovered by the ICA and PWC-ICA algorithms. Observe that PWC-ICA (1) Haar discovers fewer components that fit to dipoles with low RV values than AMICA, Extended Infomax, and FastICA. However, as shown in Figure 4, the PWC-ICA components that do fit dipoles with low RV correspond well with known source dipole positions and time courses in contrast to the other algorithms.

**Figure 6:**
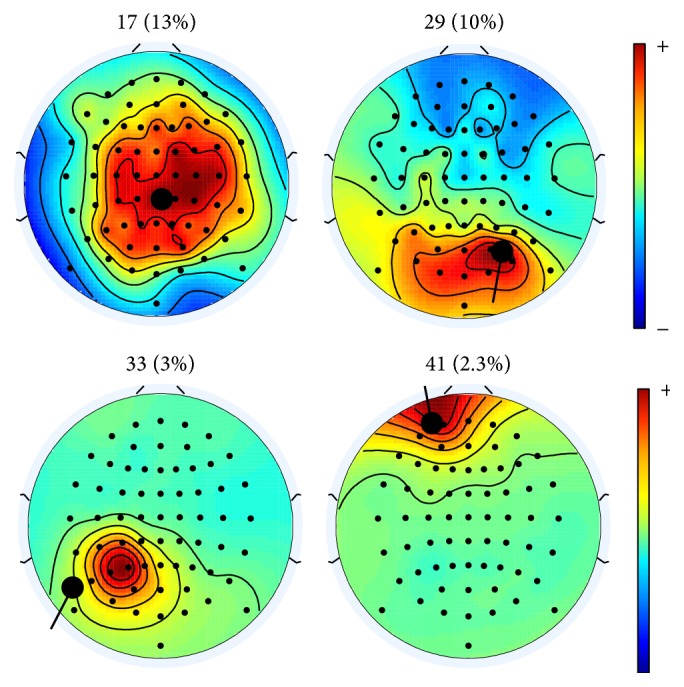
Scalp maps of the PWC-ICA (1) Haar components with the best absolute time correlations above 0.8. The fitted dipole and moment are plotted in bold black on each scalp map, and the residual variance of the fitted dipole is included in parentheses above the scalp map.

Figure 6 shows the scalp maps of the four PWC-ICA (1) Haar components of Figure 4(d) with correlations above 0.8. All four approximated sources show physically distinct behavior. Note that FastICA, Extended Infomax, and AMICA each discovered only one component that met the criteria of the four PWC-ICA (1) Haar components corresponding to the components of Figure 6.

The preceding results indicate that PWC-ICA (1) Haar returns few spurious solutions to the given simulated BSS problem. PWC-ICA (1) Haar finds 4 components that are quite highly correlated (with correlation coefficients greater than 0.8) and have physically plausible dipoles (RV < 15%) that are relatively close to the physical locations of the generating sources with which they correlate. The other tested algorithms find only one such component in each case.

More importantly, FastICA, Infomax ICA, AMICA, and the proposed Hilbert approach find many components with good dipole RV values but that do not correlate well with a source and/or are not physically close to a source they correlate well with. Often in real-world ICA analysis on EEG, the sole indicator of the quality of a BSS solution is the RV and physical locations of the associated dipoles. Without prior knowledge of the generated sources, one cannot validate an unsupervised ICA solution. PWC-ICA (1) Haar returns fewer “good” dipoles as measured by RV, but all of the PWC-ICA dipoles match reasonably well to a generating source, whereas most of the “good” dipoles from FastICA, Extended Infomax, the complex Hilbert approach, and AMICA do not correspond well to actual generating sources.

The above analyses show that for coupled oscillators good RV values from dipole fitting do not always correspond to good and distinctive representations of the sources underlying signal data. By extension, fitting more components to dipoles in the case of EEG data is not always an indicator of a better solution to the BSS problem. Great care must be exercised if one is to make definitive statements about the physical plausibility (and by extension, reality) of a discovered component.

The BSS problem in the above experiment required fitting an overcomplete basis in the signal space relative to the actual number of generating sources. This is an appropriate scenario, because the number of sources in real data is always unknown. However, to isolate the effect of overcompleteness, we randomly sampled 13 signal channels from the set of 64 and sought a 13 × 13 demixing solution using ICA and PWC-ICA algorithms. We found no significant differences in the discovered solutions across algorithms after 20 iterations of the experiment.

## 5. Mutual Information Reduction and Dipoles

Delorme et al. [9] have developed a comprehensive series of tests of the mutual information reduction (MIR) and pairwise mutual information (PMI) as well as the quality of dipole fitting via residual variance (RV). They report a meaningful linear relationship between mutual information reduction (MIR) given by a particular algorithm and the percentage of dipoles fit falling below a certain threshold RV. They also released MICA, a collection of datasets used to calculate these relationships. The MICA package contains a MATLAB toolbox to perform these calculations, enabling comparisons by other researchers. We used this package to compare BSS and EEG dipole fitting results for the PWC-ICA algorithms.

The MICA release includes 14 datasets collected from different subjects in the same experimental paradigm. Each dataset consisted of an EEG recording over 71 channels from an evoked response potential experiment, sampled at 250 Hz and partitioned into 1.4 second windows (Delorme et al., 2012) [9]. Each dataset included approximately 700 epochs, resulting in approximately 250,000 vector observations in *ℝ* ^71^ (or *ℂ* ^71^ for the complex FastICA of PWC-ICA), excluding the loss of some number of epoch endpoints depending on *h*. We used the legacy version of DIPFIT packaged in the MICA release to find the best dipole fit for each of the 71 sources. We refer the reader to the documentation and references associated with the MICA release for more details.

Delorme et al. report that one of the 14 subjects had poor ICA decompositions from AMICA and Infomax and excluded this subject from their analysis. However, since several PWC-ICA algorithms (as well as Pearson ICA) returned a significant number of components that fit to physiologically plausible dipoles on this dataset, we included this subject in our analysis. In addition to some ICA approaches discussed in the introductory section, we also included results from Delorme et al. [9] on the same experimental datasets using AMICA [39].

**Figure 7:**
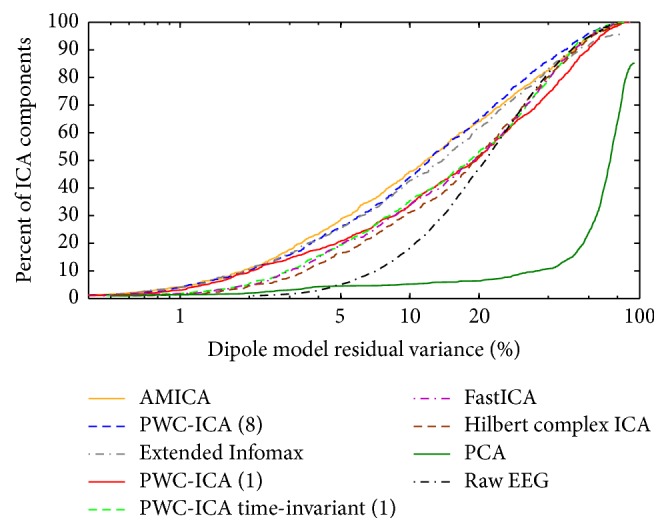
Percent of discovered components that fit a dipole with RV falling below a threshold value (horizontal axis). PWC-ICA (*h*) refers to a step size of *h* used in the construction of the pairwise dataset.

We calculated PWC-ICA decompositions for each of the 14 datasets in the MICA release data, with varying time scales specified by step sizes. We fit dipoles to each discovered independent component for each subject and algorithm, recording RV values ranging from 0 to 100 percent for each dipole.  Figure 7 compares the cumulative distribution of dipole residual variance for various ICA algorithms, similar to Figure 4(a) of Delorme et al. [9].

PWC-ICA (8) has comparable performance to AMICA and Extended Infomax in terms of dipole fitting by quantity and quality (RV percentages). Paired Welch's *t*-tests comparing the pooled RV distributions of PWC-ICA (8) with AMICA and Extended Infomax return *p* values of 0.60 and 0.61, respectively. PWC-ICA (1) fits dipoles at a similar rate to AMICA and Extended Infomax at low RVs but drops to the PWC-ICA (1) Haar and FastICA rates for dipole fitting by RV above 4 percent RV. The time-invariant PWC-ICA (1) Haar components fit to a relatively smaller number of quality dipoles, comparable to the dipole fitting characteristics of FastICA.

We also calculated the mutual information reduction (MIR) of each discovered transformation relative to the signal data. Mutual information in this context is defined as the difference between the component entropies of the channels of a multidimensional signal and the joint entropy of all channels. While the mutual information of a high dimensional signal is prohibitively expensive to compute explicitly, Delorme et al. [9] describe a procedure to compute the mutual information reduction under a linear transformation of a multivariate system. The goal of ICA solutions to the BSS problem can be thought of in the context of mutual information: a reduction in the mutual information carried across the components of a multivariate signal would indicate that the components have become more independent of each other and hence better isolate the presumably independent source behavior.

**Figure 8:**
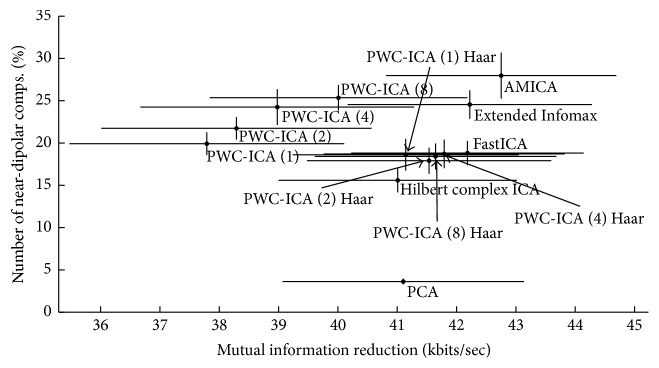
Percent of independent components whose dipole fits fall within a 5% RV threshold versus MIR by algorithm, averaged over 14 subjects. Horizontal and vertical bar indicate standard error of the mean for MIR and percent dipoles fit, respectively.

Figure 8 plots the average percentage (across 14 subjects) of discovered components that fit dipoles with RV less than or equal to 5% versus the average MIR of each tested algorithm. Delorme et al. [9] found a linear relationship between average MIR and this average percentage of well-fitting dipoles among various ICA algorithms. We observe that PWC-ICA methods may not fit this linear relationship. This is consistent with our understanding of the methodology. PWC-ICA seeks an ICA solution to the BSS problem in a complex vector space and reinterprets the result in signal space. Hence, we do not expect that the algorithms will maximize MIR measured in the base signal space, despite having relatively good performance at fitting physically plausible dipoles.

We also note that the time-invariant PWC-ICA (*k*) Haar algorithms tend to fit a lower number of dipoles with low RVs. However, as we observed in Section 4.2, this may not necessarily indicate that the performance of the method as a BSS solution is deficient. Again, recall that in the case of our simulation with a realistic forward head model, PWC-ICA (1) Haar found only a few components that fit to “good” dipoles, but all of those components actually matched a generating source well. In contrast, the alternate algorithms found many more “good” dipoles by RV that nonetheless poorly matched the known sources. Thus, we caution against the common assumption that more good fitting (in terms of RV percentages) dipoles indicate a better BSS solution.

**Figure 9:**
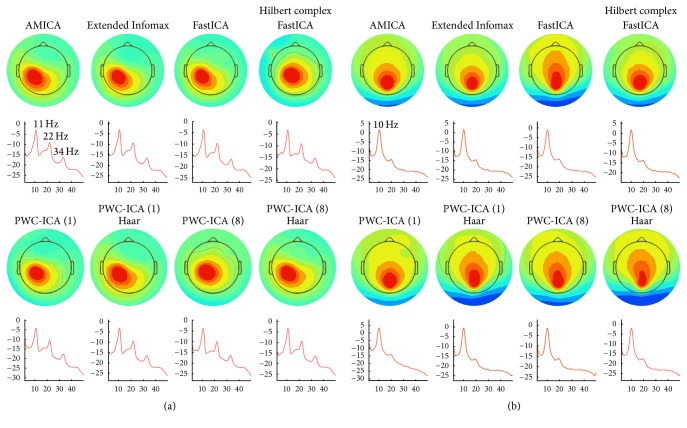
Single components corresponding to (a) left mu rhythm and (b) alpha band activity in subject 2 of the datasets provided by Delorme et al. [9]. The top plot in each case depicts a scalp map associated with the component, and the bottom plot is the mean activity spectra, with the horizontal axis measuring frequency (Hz) and the vertical axis measuring power in units of 10 log_10_ *μ*V^2^/Hz.

We are also interested in verifying that PWC-ICA component time courses represent recognizable neural processes. Similar to Delorme et al. [9], we present evidence that is indicative of broader patterns we see in the behavior of PWC-ICA.  Figure 9 displays the details of particular components found in a single subject across eight different ICA and PWC-ICA algorithms that correspond to left* mu* rhythm activity and alpha wave activity, respectively.

## 6. Discussion

Motivated by the desire to incorporate time information into ICA, this work introduces Pairwise Complex ICA (PWC-ICA), a blind source separation method that accounts for the sequential ordering of data in separating sources. Our approach, which maps a signal and its rate of change into a complex vector space, imposes additional stationarity and smoothness constraints on the solutions. The PWC-ICA method performs source separation in the mapped space and then remaps the resulting demixing matrix into the original signal space in a well-defined manner.

Briefly summarizing the comparative results between PWC-ICA and the alternate tested approaches including FastICA, Extended Infomax, AMICA, and the proposed Hilbert complex ICA approach, we found that instances of PWC-ICA performed better than the tested existing approaches in 2 of the 3 simulations of random mixing in Section 4.1 and in the simulation coupled with a realistic forward head model in Sections 4.2 and 4.3. In Section 5, all PWC-ICA approaches found interpretable BSS solutions on real EEG data that were comparable to many of the existing tested BSS approaches.

The most dramatic evidence for the usefulness of PWC-ICA was the performance of PWC-ICA (1) Haar in the case of the simulation using a realistic forward head model. In this case PWC-ICA (1) Haar found a relatively small number of good dipoles as measured by residual variance, but all of those dipoles fit to the (known) sources reasonably well. On the other hand, the alternate approaches found many good dipoles that did not correspond well to the known generating sources. This flies against much conventional wisdom in the use of ICA in EEG settings, where a larger number of “good” dipoles may be associated with a better source separation solution. Without prior knowledge of the actual sources, such an assumption may not produce reliable results. Because we directly impose the assumption of stationary mixing in our proposed formulation, the resulting demixing solutions may differ from alternate ICA approaches by finding fewer ICs that are more likely to correspond to well-behaved and persistent sources. There are several further lines of reasoning that we touch on below regarding why PWC-ICA may give different but meaningful results relative to the status quo of ICA algorithms.

Because the PWC-ICA method uses rate of change information, the transformation has an intrinsic time scale, which results in families of transformations for different embeddings and scalings. We considered approximations based on the physical sampling rate of the intrapair interval in order to interpret these vectors as true rates of change. These approaches generally fared poorly in our simulations with prior knowledge of sources (see Section 4.1) relative to the time-invariant mapping versions of PWC-ICA, which can be identified with simple Haar wavelet transforms on observation pairs. However, it is worth noting that the time-variant approaches that performed poorly in simulations did result in higher numbers of fitted dipoles by RV in real EEG data and broke the linear relationship between MIR and dipole fit percentage that was proposed by Delorme et al. [9]. As mentioned before, we caution against inferring performance from percentage of dipoles fit in this case.

The time-invariant strategies (denoted by PWC-ICA (*h*) Haar for different pairwise intervals *h*) in Experiments 1 and 3 in Section 4.1 and the forward head model simulation in Sections 4.2 and 4.3 performed better (either by Amari index or number of recovered sources) than both the time-inclusive (PWC-ICA (*h*)) and classic algorithms such as AMICA, Extended Infomax, and FastICA.  Experiment 3 and the realistic forward head model simulation involved coupled oscillators of varying degrees of complexity. The coupling no doubt violates some part of the assumption of “independence.” However, if source separation is to be applied beyond separation of artifacts to understanding of brain dynamics, it must almost certainly be able to capture coupled sources. Interestingly, the PWC-ICA (1) Haar model was able to capture some of the uncoupled sources (e.g., sources 11 and 12 in the dynamically coupled model) more closely than any of the other methods.

Our results on a simulation with a realistic forward head model show that AMICA, Extended Infomax, and FastICA found many “good dipoles” that* did not *correspond to the actual sources. In contrast, PWC-ICA (1) Haar found few “good dipoles” with low residual variance, but all of these dipoles corresponded to actual sources. This result indicates that researchers should be cautious in assuming that all “good” dipoles correspond to real sources and should not use the number of “good” dipoles found as a sole measure of the quality of an ICA solution.

The particular coupled oscillator model used with the forward head model in Section 4.2 had natural clusters of sources. Multidimensional ICA [40] and flavors of independent subspace analysis (ISA) [41, 42] may be better suited to solving such BSS problems. The aforementioned ISA approaches, however, rely on prior knowledge of source subspace dimensionality, that is, the number of sources in coupled clusters. We speculate that PWC-ICA may achieve good solutions as an indirect consequence of minimizing mutual information in the complexification of the dynamic phase space without relying on prior assumptions about the dimensionality of meaningfully independent subspaces of the signal space.

In analysis on real signals, the time-inclusive PWC-ICA (*h*) methods generally found more dipoles and displayed higher cross-subject consistency than the time-invariant methods. Since we do not know the actual sources of real signals, we cannot determine which of the discovered dipoles fit well to real sources for any method. The results presented in Section 5 on real data should not be interpreted as a measure of algorithm performance but are meant to illustrate the behavior of various approaches in real experimental contexts. The component scalp maps and component time courses show similar behavior across all of the PWC-ICA methods as well as AMICA, Extended Infomax, and FastICA.

There is a close relationship between sampling frequency and the time scale of the rates of change captured by the PWC-ICA (*h*). Our experiments show that the dipole RV distributions and MIR of PWC-ICA (8) on the MICA release subjects' data from Section 5 closely resembled the dipole RV distributions and MIR of PWC-ICA (4) on the same signals downsampled by a factor of two. Additionally, the frequency response characteristics of PWC-ICA (8) relative to alternate PWC-ICA versions for the sampling rate used may favor the discovery of physiologically meaningful sources in the upper “alpha” (8–12 Hz) and/or “beta” (>12 Hz) bands [43], as may be evidenced by the high number of dipoles fit by PWC-ICA (8) in Figure 7 (with the appropriate caveats about dipole fitting not necessarily indicating a good solution). Specifically, the PWC-ICA (8) mapping into the imaginary part of the complex vector is a convolution of the signal with a filter [1,0, 0,0, 0,0, 0,0, −1]/(Δ*t*). The first local maximum in this filter's impulse frequency response occurs at 15.6 Hz for sampling rate 250 Hz. Thus PWC-ICA (8) in this experiment may isolate signal features in the upper alpha and beta bands corresponding to synchronous beta band activity in the sensorimotor and frontal cortices [44].

From a holistic perspective, describing PWC-ICA as an ICA method may be a misnomer. PWC-ICA does not return maximally independent components by information-theoretic measures in the signal space. Rather, PWC-ICA finds components with dynamics that are more independent according to information-theoretic measures in a complex space that incorporates the dynamics of the system. The PWC-ICA family of algorithms generally has lower mutual information reduction in signal space than Extended Infomax or AMICA. However, as illustrated by Figure 8, mutual information reduction has large variability across subjects and may not be the most reliable way to assess performance.

An important consideration in our development of PWC-ICA was the effect of the proposed differencing operation on the signal-to-noise ratio of the transformed data. Indeed, in the case where desired source behavior varies slowly relative to a fixed sampling rate, differencing of the data will result in a dramatic decrease of the power of such a source relative to the power of Gaussian noise, which remains invariant with respect to **V**_Haar_. As the frequency of an oscillating source increases and approaches the Nyquist frequency, the differences are actually better able to capture meaningful information relative to noise than the sums.

When viewed as a convolution operator on signals, **V**_Haar_ applied to pairs separated by a lag of *h* = 1 divides the signal into two bands overlapping at half the Nyquist frequency. As *h* increases, the widening convolution induced by **V**_Haar_ serves to divide the signal into an increasing number of alternating bands. Thus, we can understand the mapping of pairwise vectors to a complex vector space as sorting signals into some number of bands (depending on *h*), where alternate bands are mapped into either the real or imaginary part of complex vectors.

Taking these points into consideration, the good performance of PWC-ICA (1) Haar in the case of the realistic forward head model simulation of Section 4.2 may be due to a confluence of two factors. First, as mentioned above, the choice of lag *h* = 1 may be fortuitous for the particular system based on the natural frequencies of the generating sources. Second, the transformation **V**_Haar_ used in the Haar approach commutes with the complex linear structure used in the complexification of the phase space and thus has a consistent representation in the complex space itself.

Another factor in considering the applicability of PWC-ICA methods is performance. In theory, PWC-ICA could be used in conjunction with any complex ICA implementation, and our publicly released implementation allows users to provide their own implementation. The results reported in this paper use the FicaCPLX extension of complex FastICA developed by Koldovský and Tichavský. We have found the saddle-point heuristic used by this implementation to be very stable and fast. The PWC-ICA operations themselves are of trivial computational complexity relative to solving the BSS problem in a complex vector space via complex ICA; thus performance is highly dependent on the chosen complex ICA implementation. PWC-ICA provides a framework for mapping real signals into a complex vector space and reinterpreting a complex demixing solution as a real demixing solution in the original signal space.

In conjunction with this paper, the authors have released PWC-ICA toolbox for MATLAB that performs PWC-ICA on real, vector-valued signal data. The toolbox works either as a stand-alone method or as an EEGLAB extension with a GUI interface. The PWC-ICA toolbox is available at https://github.com/VisLab/pwcica-toolbox, in addition to scripts and data to reproduce the experiments in this paper.

## Figures and Tables

**Figure 10:**
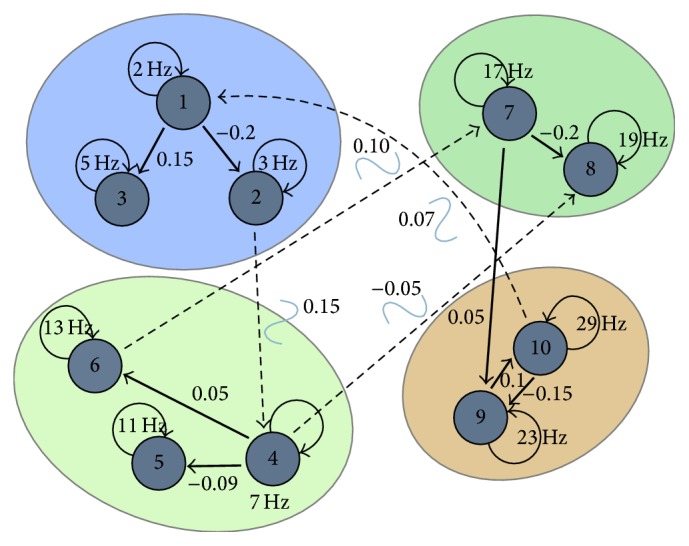
A graphical depiction of coupling dependencies in the 10-source vector AR(2) model. Each circle represents a generated source modeled by a damped harmonic oscillator with a given fundamental frequency, randomized damping coefficients, and coupling terms depicted by the connecting lines. Solid black lines indicate a static first-order dependency with coupling coefficient denoted in the direction of the arrow. Dashed lines indicate a second-order dependency. The second-order couplings vary dynamically according to a sinusoidal function with frequencies varying between 0.1 and 1 Hz and amplitudes denoted in the figure.

**Table 1:** Comparison of Amari indices for different BSS approaches trained on randomly mixed VAR simulated data. The algorithms with average Amari indices significantly lower (*p* < 0.05) than AMICA, Extended Infomax, and FastICA are bold, with the largest *p* value for the three comparisons shown in parentheses. The cells that are not bold had Amari index distributions that were not significantly lower (*p* < 0.05) than at least one of the algorithms AMICA, Extended Infomax, and FastICA. The average baseline was computed by generating 50,000 random demixing matrices for each of the 20 mixing matrices, resulting in a distribution of one million Amari indices, the average of which is reported.

BSS algorithm	Amari indices		
	Experiment 1: static uncoupled oscillators	Experiment 2: static coupled sources	Experiment 3: dynamic coupled oscillators
AMICA	0.31	0.37	0.25
Extended Infomax	0.31	0.38	0.25
FastICA	0.32	0.30	0.26
PWC-ICA (1)	0.35	0.29	0.28
PWC-ICA (2)	0.34	0.41	0.26
PWC-ICA (4)	0.35	0.41	0.24
PWC-ICA (8)	0.30	0.41	** 0.23** (*p* < 2.3*E* − 3)
PWC-ICA (1) Haar	0.30	0.32	0.25
PWC-ICA (2) Haar	** 0.21** (*p* < 1.5*E* − 11)	0.35	** 0.23** (*p* < 4.5*E* − 4)
PWC-ICA (4) Haar	** 0.21** (*p* < 1.5*E* − 10)	0.40	** 0.23** (*p* < 4.4*E* − 2)
PWC-ICA (8) Haar	** 0.27** (*p* < 1.5*E* − 5)	0.38	0.24
Hilbert complex	** 0.28** (*p* < 1.5*E* − 4)	0.38	** 0.23** (*p* < 6.7*E* − 3)
Average baseline	0.36	0.42	0.36

**Table 2:** Average number of sources that are matched by a demixing solution component with absolute correlation ≥ 0.7 per experiment. Recall that Experiments 1 and 3 had 10 sources and Experiment 2 was generated by 5 sources. Bold values correspond to the significant PWC-ICA algorithms (by the Amari index) as described in Table 1.

BSS algorithm	Avg. number of sources matched (*R*≥ 0.7)		
	Experiment 1: static uncoupled oscillators	Experiment 2: static coupled sources	Experiment 3: dynamic coupled oscillators
AMICA	1.35	2.65	4.00
Extended Infomax	1.55	3.35	4.90
FastICA	1.20	5.00	4.80
PWC-ICA (1)	4.95	3.80	4.05
PWC-ICA (2)	5.50	3.15	4.00
PWC-ICA (4)	5.85	2.70	4.05
PWC-ICA (8)	2.45	2.55	**4.00**
PWC-ICA (1) Haar	1.60	3.30	3.45
PWC-ICA (2) Haar	** 5.50**	3.40	** 3.65**
PWC-ICA (4) Haar	**5.05**	2.30	** 4.20**
PWC-ICA (8) Haar	** 4.15**	2.80	4.10
Hilbert complex	**4.50**	2.85	** 3.75**

## Appendix

### A. Source Simulations in Random Mixing Experiments

Experiment 1 (uncoupled harmonic oscillators). 
Experiment 1 of Table 1 uses a second-order vector autoregressive (VAR) model (implemented in the SIFT package) to generate 10 simulated sources of data. Recall that the order of a VAR model refers to the maximum lag of the past influences on current behavior. For instance, a VAR model of order 4 includes at least one component *x*_*i*_ such that *x*_*i*_(*t*) depends on the value *x*_*i*_(*t* − 4). We generated one hundred (100) epochs, each consisting of 500 samples using a simulated sampling rate of 200 Hz. We assigned the oscillators to have prime fundamental frequencies in the range 2 to 29 and damping constants drawn randomly from a normal distribution with mean 5 and a standard deviation of 3. Table 1 compares the average performance of different approaches for twenty 10 × 10 well-conditioned mixing matrices. The autoregressive equation used to approximate the evolution of a damped simple harmonic oscillator with damping constant *τ* and (normalized) fundamental frequency *f*_0_ is

(A.1) xHt;τ,f0=2e−1/τcos⁡2πf0xt−1−e−2/τxt−2.

Each sample was generated by evolving the autoregressive equation:

(A.2) $$\begin{array}{c} x\left(\;t\;\right)=x_{H}\left(\;t;\tau ,f_{0}\;\right)+\epsilon \left(\;t\;\right), \end{array}$$

where *ϵ* is an independent, normally distributed array of residuals.

Experiment 2 (statically coupled sources). To test performance when sources are coupled, we generated 5 sources according to the third-order vector AR model found in Example 3 of Schelter et al. [45] and mixed the sources using a 5 × 5 well-conditioned random mixing matrix. Again, we generated one hundred (100) epochs each containing 500 observations at a sampling rate of 200 Hz. The system of equations is given by

(A.3) x1t=0.9x1t−1+0.3x2t−2+ϵ1t,x2t=1.3x2t−1−0.8x2t−2+ϵ2t,x3t=0.3x1t−1+0.6x2t−2+ϵ3t,x4t=−0.7x4t−3−0.7x1t−3+0.3x5t+ϵ4t,x5t=x5t−1−0.4x5t−2+0.3x4t−2+ϵ5t.

As in the previous section, we repeated the experiment 20 times (i.e., we generated and applied 20 different random mixing matrices). Table 1 compares the accuracy (as measured by the Amari index) for the different algorithms.

Experiment 3 (dynamically coupled harmonic oscillators). 
Experiment 3 explores dynamic coupling based on a 10-source model. We incorporated several constant first-order coupling terms between components in the second-order model equations as shown by solid black lines in Figure 10. Additionally, we included several second-order dynamic couplings, indicated by dashed lines in Figure 10. The second-order coupling coefficients varied according to a sinusoidal function at frequencies between 0.1 and 1 Hz. For example, according to Figure 10, the AR equation for the 3 Hz oscillator *x*_2_ at time *t* contains a nonzero contribution from the 2 Hz oscillator *x*_1_ at time *t* − 1. Likewise, the equation for the 7 Hz oscillator *x*_4_ includes a varying contribution from the 3 Hz oscillator *x*_2_ at time *t* − 2. The equations used to generate samples in Experiment 3 are

(A.4) x1t=xH,1t;τ1,f1+0.07x2t−1+0.05x3t−1+0.08sin⁡2π110fst+0.27x4t−2+ϵ1t,x2t=xH,2t;τ2,f2−0.08x1t−1+ϵ2t,x3t=xH,3t;τ3,f3+0.04x1t−1+ϵ3t,x4t=xH,4t;τ4,f4−0.07x5t−1+0.05x6t−1+0.03sin⁡2π15fst−0.78x1t−2+ϵ4t,x5t=xH,5t;τ5,f5−0.09x4t−1+ϵ5t,x6t=xH,6t;τ6,f6+0.05x4t−1+ϵ6t,x7t=xH,7t;τ7,f7+0.08x8t−1+0.05sin⁡2π17fst+1.2x1t−2+ϵ7t,x8t=xH,8t;τ8,f8−0.09x7t−1−0.04sin⁡2π18fst−0.7x4t−2+ϵ8t,x9t=xH,9t;τ9,f9−0.15x10t−1+0.05x7t−1+ϵ9t,x10t=xH,10t;τ10,f10+0.1x9t−1+ϵ10t.

In the above equations, *f*_*s*_ is the sampling rate and *τ*_*i*_ and *f*_*i*_ are the *i*th damping constant and normalized fundamental frequency. The values of these parameters are the same as those in Experiment 1.

### B. Source Simulations in Forward Model Experiment

#### B.1. Vector Autoregressive Equations for Forward Model Simulation

The simulation used to generate the source data used in Section 4.2 is derived from a 6th-order VAR equation with cross-source coupling coefficients that are activated within one of 3 stages and modulated by generalized Gaussian function of shape parameter at least 8 symmetrically distributed about the center of the stage. The equation for the generating distribution function centered at observation *t*_0_ and scaled by *σ* is given by

(B.1) $$\begin{array}{c} g\left(\;t;\sigma ,t_{0},n\;\right)=\exp\left(\;-{\left(\;\frac{\left|t-t_{0}\right|}{\sigma }\;\right)}^{n}\;\right), \end{array}$$

which is subsequently multiplied by some constant corresponding to the desired maximum amplitude.

The sampling rate for this experiment was chosen to be *f*_*s*_ = 100 Hz, and a single 5-minute trial was generated. The three stages were centered about observations *t*_1_ = 62.5*f*_*s*_, *t*_2_ = 65*f*_*s*_, and *t*_3_ = 70*f*_*s*_ (62.5, 65, and 70 seconds, resp.). Setting the scale constant *σ* = 250 (2.5 seconds) and with natural frequency constants *f*_2_ = 20 Hz and *f*_3_ = *f*_4_ = 10 Hz and damping constants *τ*_1_ = 20, *τ*_2_ = *τ*_4_ = 6, and *τ*_3_ = 7, the system of equations describing the 13-source model is given as

(B.2) x1t=2exp⁡−1τ1+100gt;σ,t1,8cos⁡2π5·x1t−1−exp⁡−2τ1+100gt;2σ,t1,8·x2t−2+ϵ1t,x2t=xH,2t;τ2,f2fs+0.01gt;σ,t1,8x3t−2+0.01gt;σ,t1,8x4t−2+1.3gt;σ,t1,8x1t−6+ϵ2t,x3t=xH,3t;τ2,f2−1fs+0.7gt;σ,t1,8x2t−2+0.1gt;σ,t1,8x4t−2+0.3gt;2σ,t2,10x5t−3+ϵ3t,x4t=xH,4t;τ2,f2+1fs+0.7gt;σ,t1,8x2t−2+0.1gt;σ,t1,8x3t−2+ϵ4t,x5t=xH,5t;τ3,f3fs+0.001gt;σ,t2,8x6t−2+0.5gt;σ,t2x3t−3+ϵ5t,x6t=xH,6t;τ3,f3fs+0.001gt;σ,t2,8x5t−2+ϵ6t,x7t=xH,7t;τ4,f4fs+1.3gt;σ,t3,8x4t−6+0.01gt;σ,t3,8x8t−2+0.01gt;σ,t3,8·x9t−2+0.01gt;σ,t3,8x10t−2+ϵ7t,x8t=xH,8t;τ4,f4fs+0.6gt;σ,t3,8x7t−2+ϵ8t,x9t=xH,9t;τ4,f4+1fs+0.6gt;σ,t3,8x7t−2+ϵ9t,x10t=xH,10t;τ4,f4−1fs+0.6gt;σ,t3,8x7t−2+ϵ10t,x11t=1.3x11t−1−0.8x11t−2+ϵ11t,x12t=1.2x12t−1−0.4x12t−2+ϵ12t,x13t=0.8x13t−1−0.4x13t−2−0.1x13t−4+ϵ13t.

#### B.2. Source Locations by Region of Interest

As described in Section 4.2, each of the 13 source activations generated in the 3-stage VAR process was assigned to a randomly selected region of interest and mapped to the scalp according to the MNI “colin27” brain [35]. Dipoles were then fed through a forward model, generating signals at simulated electrodes on the scalp of the model head. The selected regions of interest are listed here by verbatim labels of the Destrieux atlas [46]:

1. S_occipital_ant R,
2. G_and_S_subcentral R,
3. S_oc-temp_med_and_Lingual L,
4. S_calcarine L,
5. G_and_S_frontomargin L,
6. S_precentral-inf-part L,
7. S_temporal_sup L,
8. G_Ins_lg_and_S_cent_ins R,
9. S_temporal_sup R,
10. G_oc-temp_med-Parahip L,
11. G_pariet_inf-Angular L,
12. G_front_inf-Opercular R,
13. S_temporal_inf R.

Documentation of the applied forward model is available externally [31], and we have made the exact routines used to generate the data in this experiment available in an addendum to the freely available PWC-ICA toolbox.
